# Evaluation of Covid-19 anti-spike IgG antibody five months after the second Covid-19 vaccination 

**DOI:** 10.3205/dgkh000446

**Published:** 2023-09-07

**Authors:** Reyhaneh Alipoor Rafie, Leila Azimi, Shahnaz Armin, Amirali Aghamohammadi, Abdollah Karimi, Fatemeh Fallah, Hannan Khodaei, Roxana Mansour Ghanaie, Masoud Alebouyeh

**Affiliations:** 1 Department of Infectious Diseases, Shahid Beheshti University of Medical sciences, Tehran, Iran; 2 Pediatric Infections Research Center, Research Institute for Children’s Health, Shahid Beheshti University of Medical Sciences, Tehran, Iran

**Keywords:** anti-spike IgG antibody, ELISA, vaccination, healthcare worker

## Abstract

**Background:**

Studies in different communities have shown significant differences in IgG antibody titers in the time period after the first and second doses of the vaccines. This study aimed to serologically evaluate the IgG anti-spike antibody titer five months after injection of the second COVID-19 vaccine in healthcare workers.

**Materials and method:**

This study was performed in healthcare personnel for whom five months had passed since their second anti-Covid-19 vaccination. The level of IgG antibody against SARS-CoV-2 spike protein was measured by ELISA. Healthcare workers in Mofid Children’s hospital received three brands of vaccines: Sputnik V, Sinopharm, and AstraZeneca.

**Results:**

The mean titer of anti-spike IgG was 4.3±2.29 units. The percentage of positive cases of the antibody was estimated to be 96.4%. The titer of anti-spike IgG antibody was dependent on both the occupational area and a positive history of Covid-19 disease.

**Conclusion:**

About 96.4% of the staff vaccinated against Covid-19 had a high titer of anti-spike IgG antibody even five months after inoculation of the second dose. The field of occupational can affect the level of antibody present

## Zusammenfassung

**Hintergrund:**

Studien in verschiedenen Populationen haben signifikante Unterschiede bei IgG-Antikörpertitern im Zeitraum nach der ersten und zweiten Impfung gezeigt. Ziel der Studie war es, den IgG-Anti-Spike-Antikörpertiter mindestens fünf Monate nach Gabe der zweiten Dosis des COVID-19-Impfstoffs bei Beschäftigten im Gesundheitswesen serologisch zu bewerten.

**Material und Methode:**

Diese Studie wurde an medizinischem Personal durchgeführt, bei dem seit der zweiten Dosis der Anti-Covid-19-Impfung mindestens 5 Monate vergangen waren. Der IgG-Antikörper Titer gegen das SARS-CoV-2-s-Spike-Protein wurde mittels ELISA gemessen. Das Gesundheitspersonal im Mofid-Kinderkrankenhaus wurde mit drei Arten von Impfstoffen geimpft: Sputnik V, Sinopharm und AstraZeneca.

**Ergebnisse:**

Der mittlere Titer von Anti-Spike-IgG betrug 4,3±2,29 Einheiten. Der Prozentsatz der positiven Antikörper wurde auf 96,4 % geschätzt. Der Titer der Anti-Spike-IgG-Antikörper war abhängig vom Berufsfeld sowie von der positiven Vorgeschichte einer Covid-19 Erkrankung.

**Schlussfolgerung:**

Etwa 96,4 % der gegen Covid-19 geimpften Mitarbeiter wiesen fünf Monate nach der 2. Impfung einen hohen Titer von Anti-Spike-IgG-Antikörpern auf, wobei das Berufsfeld die Höhe des Antikörper-Titers beeinflussen kann

## Introduction

Inadequate control of the Covid-19 pandemic necessitated the development of a variety of vaccines against the virus. Different types of vaccines have shown high efficacy in clinical trials [1], [2]. In recent years, mRNA replacement has been used to increase the effectiveness of these vaccines. In total, the Covid-19 virus consists of four major structural components, including the spike protein, the nucleocapsid protein, the envelope protein, and the membrane protein [1], [2]. From these, IgG-neutralizing antibodies against spike proteins are produced and secreted. It should be noted that an effective vaccine against this virus is a vaccine that elicits a favorable immune response, especially against spike proteins [3]. This response is mainly in the form of the production of antibodies against spike proteins that remain strong for longer time periods, even after vaccination, indicating their effectiveness of preventing infection [4]. According to the results of some studies, the time required reach the peak level of anti-spike IgG antibody as well as the time elapsed before the antibody levels decrease depends on the type of vaccine and the previous history of infection with Covid-19. Even the demographic parameters of patients were completely different [5], [6]. However, most studies emphasized that the IgG antibody levels using almost all vaccine brands with different platforms increased dramatically after the first and second doses and usually after two weeks of vaccination up to 3 months later [7]. Many of these studies also described various factors associated with increase of antibody levels, including female gender, young age, a previous history of Covid-19 infection as stimulants, whereas antibody levels were inhibited by the use of immunosuppressive drugs or underlying malignancies [8], [9], [10]. 

Healthcare workers (HCW) are a high-risk population in terms of exposure to COVID-19, through patient interactions and the hospital environment. Thus, one of the top priorities of the World Health Organization (WHO) is to utilize COVID-19 vaccinations to maintain healthcare services and minimize the spread of infection within hospitals [11], [12]. In this regard, following up on the antibody titer status and antibody decline in this important community should be one of the main global goals. When evaluating the titer of this antibody in different populations, it is important to consider that various brands vaccine with diverse platforms are used, particularly in our society. 

This study aimed to evaluate the antibody titer and decrease in antibody levels in HCW after their second COVID-19 vaccination. 

## Materials and method

### Setting and sampling

The cross-sectional study was performed among HCW of Mofid Children’s Hospital in Tehran for whom five months had passed since their second COVID-19 vaccination. Blood samples were collected from these HCW and transferred to the laboratory of the Pediatric Infections Research Center (PIRC) to separate the blood serum with a centrifuge at 4,000 rpm for 10 minutes. 

### Data gathering and ELISA

The HCW data were collected through the completion of an information form. The sera were stored at –80°C until running the Enzyme-linked immunosorbent assay (ELISA). To measure the level of IgG antibody against the Covid-19 virus spike protein, a Euroimmune ELISA kit (Lübeck, Germany; Lot No. E200519AY) was used. The titer ratio of antibody ≥1.1 is considered an acceptable titer of antibody and positive result according to Kit protocol. 

### Statistical analysis

The results were presented as mean±standard deviation (SD) for quantitative variables and were summarized by frequency (percentage) for categorical variables. Continuous variables were compared using the t-test or Mann-Whitney U-test, whenever the data did not appear to have normal distribution or when the assumption of equal variances was violated across the study groups. The categorical variables were compared using the Chi-Square test. p-values ≤0.05 were considered statistically significant. For the statistical analysis, SPSS version 23.0 for Windows (IBM, Armonk, New York) was used.

Ethical approval was given by the Research Ethics Committees of Research Institute of Children’s Health, Shahid Beheshti University of Medical Science (No. IR.SBMU.RICH.REC.1400.059, 31 July 2021).

## Results

A total of 234 vaccinated HCW enrolled five months after the second COVID-19 vaccination. Three types of vaccines were given to the HCW: Sputnik V, Sinopharm, and AstraZeneca. The mean age of patients was 39.8±9.3 years, with a range of 23 to 60 years. 72.6% were female. The baseline characteristics of study participants are shown in Table 1 (Tab. 1). In total, 103 cases (44.0%) had a previous history of SARS-CoV-2 infection with PCR-positive or pulmonary involvement evident on CT. A history of receiving antiviral drugs was reported in 21 cases (20.3%); 2 cases (1.9%) were hospitalized at the time of the disease. 

The mean titer evaluation of anti-spike IgG antibody five months after vaccination was 4.33±2.29 units. Accordingly, the percentage of positive cases of the antibody titer was estimated to be 96.4%. As indicated in Table 2 (Tab. 2), the titer of the anti-spike IgG antibody was dependent on some baseline parameters including, occupational field and a positive history of Covid-19 disease (P≤0.05). 

The highest anti-spike IgG antibody titer was reported among physicians (5.01±2.48), followed by paramedics (4.8±2.03) and nurses (4.2±2.26). The mean titer of anti-spike IgG antibody in personnel with and without a history of Covid-19 infection was 5.3±2.08 units and 3.7±2.24 units, respectively, which was significantly different between the two groups (P<0.001). In this regard, the titer of anti-spike IgG antibody was independent of gender, age, and even the platform of vaccine (P≤0.05).

## Discussion

Evaluating the trend of changes in the antibody titer of the Covid-19 spike protein is the most important indicator in assessing the level of immunity provided by vaccination against this disease [13]. Studies in different communities have shown significant differences in IgG antibody-titer duration at different intervals after vaccination [14]. Various underlying factors related to demographic characteristics, vaccine characteristics, and clinical history of individuals have been quite effective on the level of its immunogenicity and its durability [14]. But the results of the studies were completely heterogeneous. What we addressed in the present study was the evaluation of IgG anti-spike antibody titer five months after inoculation of the second dose of Sputnik V, Sinopharm, and AstraZeneca COVID-19 vaccines and the determination of related factors among the HCW of Tehran Children’s Hospital.

One of the achievements in this study is positive cases of the presence of acceptable anti-spike IgG in 96.4% even five months after the second vaccination. Among the underlying factors related to antibody titer and positive immunogenicity response, previous history of SARS-CoV-2 infection and occupation are the most important parameters, according to our results (P≤0.05) and those of other studies [15], [16], [17]. In the study by Papaneophytou et al. [7], which was similar to ours, IgG antibody levels against spike-virus proteins were significantly higher in people with a history of previous infection who had received at least one dose of mRNA vaccine.

The effect of these two factors on the titer of the antibody can be explained. A history of SARS-CoV-2 infection induces innate immunity and antibody production. Thus, a high titer of antibodies was observed, because of the involvement of innate immunity and acquired immunity after vaccination.

Furthermore, occupation is directly related to the degree of exposure to infected people and as a result to the coronavirus. Physicians are the highest-risk group because of close, long contact with infected patients. Therefore, it was expected that this group would have high levels of antibodies.

In this regard, other underlying factors including sex, age, and even the platform of vaccine administered did not predict antibody levels (P≥0.05). Therefore, it seems that the difference between innate and natural immunogenicity between different groups of HCW seems to be completely different. The effect of a history of infection on the anti-spike antibody titer is well established; in various studies, it has been emphasized that it indicates an overlap between innate and acquired immunity against the virus [16]. 

Overall, it seems that even five months after the last vaccination, we are still experiencing high immunogenicity against Covid-19 disease, especially in the case of physicians and paramedics, as well as HCW with a previous history of Covid-19. In different studies, considering different periods for evaluating antibody titers, different demographic features, and different types of vaccines inoculated, different results have been obtained from antibody titers. Although regarding the effect of some factors such as previous history of Covid-19, antibody titers are similar in almost all studies [17]. 

Moreover, apart from the platform of vaccine, two doses of each vaccine will be associated with a much higher increase in IgG antibodies, and this level will be even higher in patients with a previous history of infection [7]. A study conducted among HCW in Italy evaluated the BNT162b2 mRNA vaccine by determinants of serological protection of COVID-19 vaccine during the nine-month period following vaccination by determining the spike-protein IgG in serum evaluating the spike-protein IgG [18]. The results of that study showed that 99.5% of the HCW who received two BNT162b2 vaccinations had positive serological results for as long as 250 days after the second dose of the vaccine [18]. That study [18] confirmed our results and showed an acceptable anti-spike in IgG titre 5 months after the second vaccination. 

In the study by Kobashi et al. [8], lower age, female gender, lack of immunosuppressive drugs, and lack of adverse reactions after the first dose of the vaccine were associated with higher levels of IgG antibodies following vaccination, which was not found in our study. In the study by Duro et al. [9], vaccinated people with a previous history of Covid-19 infection had slightly higher antibody levels than those without a history of previous infection. These results are congruent with ours. IgG antibody levels were also higher in women, but lower in the Astrazenka vaccine[9] group than in the other vaccine groups, according to the results of the study by Duro et al. [9]. In all vaccinated subjects, antibody levels were elevated for 1 to 2 weeks and peaked within 4 to 6 weeks. After that, however, IgG antibody levels gradually decreased [9], the results of which, especially regarding the decrease in antibody titer after the sixth week, were in stark contrast to the present study. Furthermore, in the study by Kaneko et al. [10], the antibody level was very high one month after vaccination, but it was not clear whether this high titer would remain strong over the following months. In the studies by Maneikis et al. [19] and Nam et al. [20], the type of vaccine was shown to influence the level of IgG anti-spike antibody, which was completely different from the results of the present study. These divergent results may be due to different conditions in different countries during the COVID-19 pandemic. 

## Conclusions

The results of our study and others showed that the differences in personal immunity system, life conditions, and brands and platforms of COVID-19 vaccines worldwide were responsible for the different antibody titers after vaccination and the decrease thereof over time.

### Limitations of this study

The number of those vaccinated with Astrazenka and Sinopharm was low in our study; Sputnik V was the main vaccine administered here

## Notes

### Competing interests

The authors declare that they have no competing interests.

### Funding

The research reported in this publication was supported by Elite Researcher Grant Committee under grant number [30846] from the Shahid Behehsti University of Medical Sciences, Tehran, Iran. 

## Figures and Tables

**Table 1:**
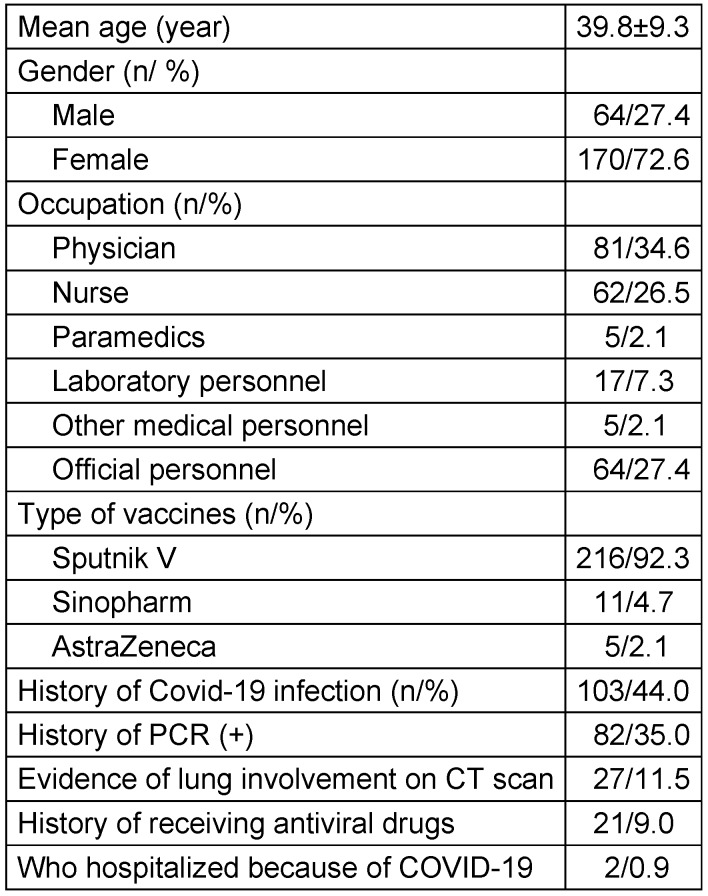
Baseline characteristics of the study population

**Table 2:**
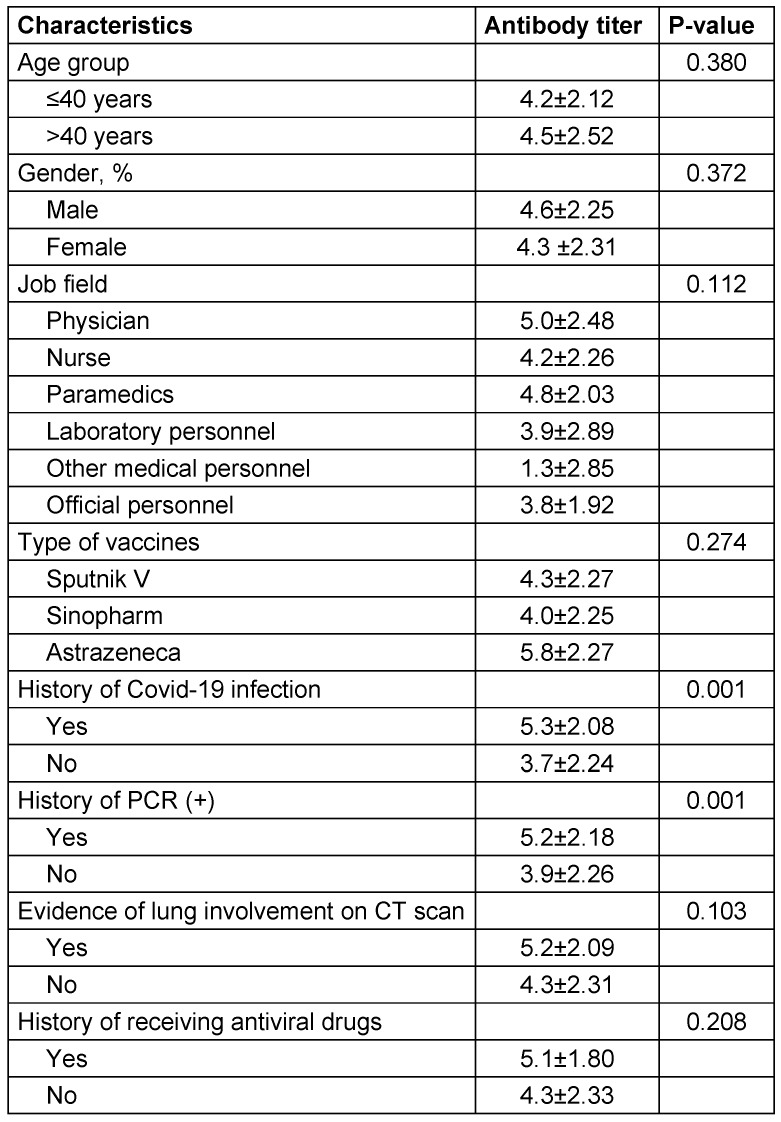
Antibody titer status in the studied HCW according to personal characteristics
